# Severe and profound hearing loss in patients with multiple sensory impairments: increased incidence of cognitive impairment

**DOI:** 10.1016/j.bjorl.2026.101778

**Published:** 2026-03-11

**Authors:** Jacob C. Lucas, Alexandra M. Arambula, Katherine Yu, Linda D’Silva, Jennifer A. Villwock, Hinrich Staecker

**Affiliations:** a Michigan Ear Institute, Farmington Hills, United States of America; b Case Western Reserve University/University Hospitals Cleveland Medical Center, Department of Otolaryngology-Head and Neck Surgery, Cleveland, United States of America; c Virginia Commonwealth University, Department of Otolaryngology-Head and Neck Surgery, Richmond, United States of America; d University of Kansas Medical Center, Department of Physical Therapy and Rehabilitation Science, Kansas City, United States of America; e University of Kansas School of Medicine, Department of Otolaryngology-Head and Neck Surgery, Kansas City, United States of America

**Keywords:** Sensorineural hearing loss, Sensory impairment, Cognition

## Abstract

**Objective:**

Determine odds of incident cognitive impairment among patients with severe to profound hearing loss and co-existing multisensory impairment.

**Patients:**

14 prospectively recruited aging (age 50+) patients with severe and profound hearing loss were pooled for analysis with 180 previously enrolled patients with demonstrated multisensory impairment. Patients were recruited as part of a cross-sectional case-control study at a tertiary care neurotologic/audiologic outpatient clinic.

**Interventions:**

Patients with severe and profound hearing loss were identified and underwent point-of-care multisensory testing and cognitive testing.

**Main outcome measures:**

Multisensory testing using the Affordable, Rapid Olfactory Measurement Array (AROMA) for olfaction, pure tone audiometric evaluations, and the Timed ‘Up and Go’ test for gait and balance. Cognitive impairment was assessed via the Montreal Cognitive Assessment for the Hearing Impaired (HI-MoCA).

**Results:**

A total of 194 patients were included. 34% (n = 66) screened positive for cognitive impairment. Olfactory dysfunction, gait impairment, and sensorineural hearing loss were all significantly (p < 0.05) associated with higher odds of cognitive impairment (ORs = 3.17, 3.71, and 3.23, respectively in a multivariate model). Subjects with dysfunction in all domains were at highest risk for cognitive impairment (OR = 15.2, p < 0.001) compared to impairment in 2 domains (OR = 5.09, p < 0.001). Severe and profound hearing loss had higher odds (OR = 8.32) compared to mild-moderately severe hearing loss (OR = 2.81) of having incident cognitive impairment.

**Conclusions:**

Dysfunction of the olfactory, auditory, and balance systems is associated with significantly increased odds of cognitive impairment. Patients with severe and profound hearing loss were more likely to have cognitive impairment.

## Highlights

• Combined sensory dysfunction increased the odds of cognitive impairment.
• More severe hearing loss increased the likelihood of cognitive impairment.
• Patients with hearing loss should be screened for other sensory disorders.

## Introduction

Cognitive Impairment (CoI) and dementia have been repeatedly linked to Sensorineural Hearing Loss (SNHL).^1^, ^2^, ^3^ Impairment of other sensory domains such as olfaction and vestibular function are also strongly linked to cognition deficits.^4^, ^5^, ^6^, ^7^, ^8^ A recent prospectively recruited case-control study examined the interplay between loss of hearing, balance, and olfaction; patients with deficits in these domains were more likely to present with a worse cognitive screening score on the Montreal Cognitive Assessment (MoCA).^9^ Additionally, deficits were additive: patients with impairment in more than one domain had an increased odds of having incident CoI. One weakness of that study was a lack of enrolled patients with severe and profound hearing loss. The resultant data was under-powered to stratify odds of CoI by hearing loss severity. Recent population studies have suggested that not only the severity of hearing loss but also the quality of rehabilitation affects dementia risk.^10^^,^^11^

Due to this, an additional cohort of patients was recruited from a population of patients undergoing evaluation for Cochlear Implantation (CI), to specifically target patients with severe and profound SNHL. 14 additional patients with severe or profound SNHL were recruited, consented, and pooled for analysis with the previously enrolled 180 patients. We determined the following research question: Among a prospectively recruited cohort of patients with variable deficits in hearing, balance, and olfaction, does worse hearing equate to an increased Odds Ratio (OR) of CoI on the MoCA screening instrument? The hypothesis was that patients with Multisensory Impairment (MSI) and severe or profound hearing loss would have a higher proportion of CoI compared to patients with either normal hearing or hearing in the mild-moderately severe range.

## Methods

### Patients

A previously described cohort of 180 patients was recruited from February 2021 through June 2021 and included “aging” above 50-years-old. Patients presenting with a chief complaint of “hearing loss” were eligible.^9^ Exclusion criteria were patients younger than 50, progressive Central Nervous System (CNS) disease, nonambulatory, unable to follow written or verbal instructions due to cognition or language barriers, conductive hearing loss, tympanic membrane perforation, hearing loss due to vestibular schwannoma, and recent COVID-19. An additional 14 patients meeting inclusion and exclusion criteria, and with documented severe or profound SNHL were recruiting during a period spanning from September 2021 through January 2021. A total of 194 patients were pooled for statistical analysis and stratified based on severity of SNHL.

## Interventions

All study-related activities were conducted with local Institutional Review Board approval. The 14 patients with severe-profound hearing loss were recruited while undergoing evaluation for possible cochlear implantation. This population was targeted due to the high rate of severe and profound sensorineural hearing loss present.

### Hearing

Hearing was evaluated based on the ANSI/AS S3.21-2004, and thresholds were recorded at 0.25, 0.5, 1, 2, 3, 4, 6, and 8 kilohertz (kHz).^12^^,^^13^ Pure-tone averages were calculated using 0.5, 1, 2, and 3 (or an average of 2 and 4 when unavailable) kHz as recommended by the AAO-HNS.^14^^,^^15^ Word recognition scores were recorded. Air conduction levels were used.

### Balance

Point of care balance and gait testing was performed using the Timed ‘Up & Go’ (TUG) instrument,^16^^,^^12^ a timed measure of gait and coordinated standing which has been previously validated in elderly populations and patients with vestibular disorders, with up to an 80 percent sensitivity in determining fall risk in these populations.^17^^,^^18^ The test is easy for research personnel to administer and acts as a proxy for both peripheral and central vestibular function, as it requires visual, proprioceptive, and vestibular inputs to perform the coordinated task. Testing for specific vestibular end-organ disease processes was not a goal of the present study, so the TUG was an easy and proven instrument to bimodally stratify patients into “impaired” and “unimpaired” balance categories based on a cutoff score of 11 seconds for the task.

### Olfaction

The Affordable, Rapid, Olfactory Measurement Array (AROMA) test is an essential oil-based instrument for characterizing degree of olfactory impairment, with validation among patients with cognitive impairment, dementia, and Alzheimer’s disease^19^^,^^20^ which has strong association with olfactory impairment.^21^ A full accounting of the olfactory testing methodology is available in previous works.^9^ AROMA was utilized to stratify patients into impaired and unimpaired olfaction categories based on patient responses to 14 scents at 4 consecutive increasing concentrations.

### Main outcome measures

The Montreal Cognitive Assessment (MoCA) has been shown to detect mild cognitive impairment at a higher sensitivity than the Mini Mental Status Examination (MMSE) and tests multiple domains of cognition including working memory, visuospatial abstraction, and recall.^22^^,^^23^ A modification for the Hearing Impaired (HI), the HI-MoCA, provides written rather than spoken instructions to prevent the hearing impairment itself from contributing to the cognition score.^24^ HI-MoCA was used in the assessment of patients with severe and profound hearing loss. A score of 26 (out of 30) was used as the cutoff for CoI. A correction for education level of 1 point was added for patients who did not complete high school.

### Statistical analysis

Data analysis was performed using the *R* statistical computing program in RStudio^25^; with the Tidyverse suite of packages used for data wrangling and transformation.^26^ The ggplot2.^27^ and audiometry^28^ packages were used to generate visualizations of the data. Univariate analysis was performed on demographic and predictor variables to evaluate their effect on the outcome variable – cognitive status. Wilcoxon Rank Sum, Pearson’s Chi-Squared, and Fisher’s exact test were used for univariate testing. Cognitive status was classified as a binary outcome; MoCA scores 26 or greater were considered normal and scores less than 26 were considered CoI. Due to this, binary logistic regression was used for multivariate analysis, using a stepwise selection procedure for variables. Odds Ratios (OR) with Confidence Intervals (CI) were calculated for predictor variables.

## Results

A total of 194 patients were included; 180 were enrolled during the initial study period, with an additional 14 individuals with severe or profound hearing loss recruited for the present study and pooled for analysis. The recruitment and testing protocols were identical for both groups. Demographic and univariate analysis of predictor variables are reported in Table 1. Of the enrolled subjects, 66 screened positive for CoI as defined by MoCA score <26. All sensory domains were individually significant contributors toward an increased odds of incident CoI. Education was also a significant predictor toward higher scores on the screening instrument and was therefore included in multivariate analysis.

**Table 1:** Univariate analysis for demographic and predictor variable inputs.

Characteristic	Normal (n = 128)^a^	CI (n = 66)^a^	p-value^b^
Age at Enrollment	66 (59, 72)	69 (63, 75)	0.094
Age Category			0.5
50‒65	53 (41%)	24 (36%)	
65+	75 (59%)	42 (64%)	
**Sex**			0.3
Female	69 (54%)	30 (45%)	
Male	59 (46%)	36 (55%)	
Education Level			<0.001
Completed College	49 (38%)	19 (29%)	
Completed High School	37 (29%)	38 (58%)	
Completed Graduate/ Professional Degree	39 (30%)	7 (11%)	
Did not complete High School	3 (2.3%)	2 (3.0%)	
Timed Up-and-Go	9.1 (8.1, 10.5)	10.4 (8.8, 13.9)	0.001
PTA (500, 1k, 2k, 3k Hz)	29 (21, 40)	39 (27, 52)	<0.001
AROMA Score	75 (66, 84)	66 (54, 73)	<0.001
Gait Status			<0.001
Normal Gait	107 (84%)	36 (55%)	
Gait Impairment	21 (16%)	30 (45%)	
Hearing Status			<0.001
Normal Hearing	58 (45%)	12 (18%)	
SNHL	70 (55%)	54 (82%)	
Hearing Severity			<0.001
Normal	58 (45%)	12 (18%)	
Mild ‒ Moderately Severe	66 (52%)	45 (68%)	
Severe ‒ Profound	4 (3.1%)	9 (14%)	
Olfactory Status			<0.001
Normal Olfaction	60 (47%)	11 (17%)	
Hyposmia	68 (53%)	55 (83%)	
Number of Sensory Losses			<0.001
0 or 1	77 (60%)	13 (20%)	
2	41 (32%)	31 (47%)	
3	10 (7.8%)	22 (33%)	

a Median (IQR); n (%).

b Wilcoxon rank sum test; Pearson's Chi-Squared test; Fisher's exact test.

**Fig. 1:**
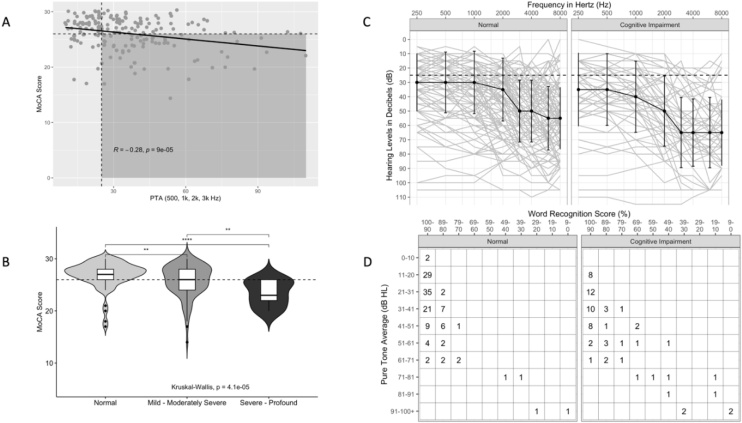
An aggregation of audiometry and hearing data for normal and CoI subjects. (A) PTA is presented on the x-axis with response variable MoCA score on the y-axis. Shaded area bounded by cutoff scores for normal MoCA (26) and normal PTA (<25 dB HL). Solid line represents best fit for Pearson Correlation; *R* and p-values are listed. (B) Violin boxplots are presented for 3 hearing categories: “Normal”, “Mild-Moderately Severe”, and “Severe-Profound. Dashed line is MoCA cutoff for normal. * p < 0.05; ** p < 0.01; *** p < 0.001; **** p < 0.0001. (C) Composite audiograms for Normal and CoI data, solid dark line is the median for each threshold, error bars correspond to 1 standard deviation. (D) AAO-HNS minimum reporting standards for raw data of PTA plotted against Word Recognition Scores (WRS).^10^

Hearing results are visualized in Fig. 1. Aggregation of the audiometric data (Fig. 1a) shows a negative correlation between MoCA score and PTA (*R* = -0.28, p < 0.001). Fig. 1b shows a clear difference between “Normal”, “Mild-Moderately Severe”, and “Severe-Profound” hearing groups; as hearing worsens, the average MoCA score within groups trends downward. Composite audiograms are shown in Fig. 1c for the Normal and CoI comparison groups. The AAO-HNS recommended reporting of hearing outcomes with PTA/WRS scattergram is shown in Fig. 1d.

**Fig. 2:**
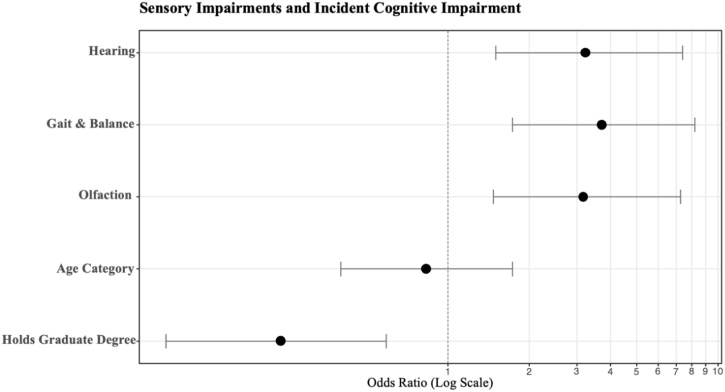
Odds Ratios for each sensory domain visualized. When hearing is represented as a binary outcome (Normal or SNHL), the odds of an incident CoI is 3.23.

Multivariate analysis is summarized in Table 2 and visualized in Fig. 2 as a plot of the Odds Ratios with Confidence Intervals. Each individual sensory deficit was found in the model to contribute to an increased odds of incident CoI (ORs = 3.17, 3.71, and 3.23 for olfactory, gait, and SNHL respectively). Sensory deficits were additive; impairment in all 3 domains was most likely to have CoI (OR = 15.2, 95% CI [5.66, 44.2], p < 0.001) compared to 2 domains (OR = 5.09, 95% CI [2.36, 11.6], p < 0.001).

**Table 2:** Binary Logistic Regression performed and listed by sensory impairment. Hearing is represented with 3 stratified groups based on severity of hearing. Worse hearing equates to a higher odds of incident CoI. Education had a protective effect on classification as CoI.

Characteristic	OR	95% CI	p-value
Hearing Severity			
Normal	‒	‒	
Mild ‒ Moderately Severe	2.81	1.28, 6.53	0.012
Severe ‒ Profound	8.32	2.11, 38.2	0.004
Gait & Balance			
Normal Gait	‒	‒	
Gait Impairment	3.57	1.65, 7.93	0.001
Olfaction			
Normal Olfaction	‒	‒	
Hyposmia	3.31	1.51, 7.72	0.004
Age Category			
50 ‒ 65	‒	‒	
65+	0.90	0.43, 1.91	0.8
Holds Graduate Degree			
No Graduate Degree	‒	‒	
Graduate Degree	0.25	0.09, 0.62	0.005

OR, Odds Ratio; CI, Confidence Interval.

When stratifying hearing status within cognitive outcomes, severe and profound SNHL were most likely to occur alongside CoI (OR = 8.32, 95% CI [2.11, 38.2], p < 0.001) compared to mild – moderately severe HL (OR = 2.81, 95% CI [1.28, 6.53], p < 0.001).

## Discussion

Impairment of multiple sensory domains seems to increase the likelihood of a co-existing cognitive deficit, at least when screening with the MoCA instrument. More interesting, in the present study, poorer hearing seems to correlate with a worse MoCA score. A worsening risk of cognitive impairment was also demonstrated in a large cohort of insurance data.^10^ Combination of balance dysfunction with visual loss has also been demonstrated to increase the risk of cognitive impairment.^29^ A number of hypotheses have been proposed to explain the link between sensory system disturbance and cognitive impairment including depletion of cognitive reserve, direct effects and loss of social interaction.^30^ Having multiple impairments could affect any of these potential mechanisms making comprehensive evaluation of our patients of paramount importance. The findings here underscore the groundwork laid in our previous work and address the under-powering of our previous study to demonstrate a relationship between worsening hearing and cognitive status. The findings here also add to the growing body of research linking hearing loss in the elderly with increased lifetime risk of dementia. The Lancet Commission has identified hearing loss as the most important modifiable risk factor for dementia.^31^ Since recent studies demonstrate cochlear implantation gives a greater improvement in dementia risk compared to amplification,^11^^,^^32^ rigorous assessment of the severity of hearing loss is an important component of patient care.

Despite these findings, the study is not without limitation. There is likely a component of selection bias; only a small percentage of recruited individuals consented to participate, and those could possibly have skewed towards having a pre-existing cognitive deficit. Our MoCA score cutoff likely over-estimates true cognitive impairment and is not sufficient alone for the diagnosis of dementia. Similarly, dedicated vestibular testing might have yielded more specific insights into the contribution of the peripheral vestibular system compared to the TUG instrument. However, the testing in this study was chosen for convenience and in the hopes of yielding some insight into cognition and its complex relationship with peripheral multi-sensory inputs. These sensory losses, especially hearing, are ripe targets for rehabilitative efforts. Future studies will attempt to alter the disease course of dementia by addressing these sensory deficits early on. The patients in this study were recruited based on complaints of hearing loss. With sensorineural hearing loss being the most common neurodegenerative disorder in man, the current study suggests that we should also screen patients who present for isolated sensory complaints for other areas of dysfunction since there is increasing evidence that rehabilitation of sensory deficits can mitigate the risk of cognitive decline.

## Conclusion

Impairment of multiple senses increases the risk of having an abnormal MoCA score. The risk of an abnormal MoCA score, and thereby higher risk of dementia is increased with worsening hearing loss.

ORCID ID

Jacob C. Lucas: 0000-0002-5441-6576

Alexandra M. Arambula: 0000-0002-5624-3613

Katherine Yu: 0000-0002-2867-5291

Jennifer A. Villwock: 0000-0001-5645-4210

## Disclosures

JAV discloses intellectual property and a filed patent (17/281121 – “Olfactory Diagnostic and Training Kits and Methods”) related to the objective olfactory testing methods used in this research.

## Data availability statement

The authors declare that all data are available in repository.

## Declaration of competing interest

The authors declare no conflicts of interest.
